# A comprehensive overview of fish envenomation and venom toxicity in Brazil

**DOI:** 10.1590/1678-9199-JVATITD-2024-0061

**Published:** 2025-05-19

**Authors:** Mônica Lopes-Ferreira, Felipe Justiniano Pinto, Yasmin Stefanie Oliveira Costa, Alessa Aparecida Burgarelli, Louise Lene Gomes Lima, Bibiana da Silva Marques, Carla Simone Seibert, Elineide Eugenio Marques, Patrícia Charvet, Vidal Haddad, João Gabriel dos Santos Rosa, Geonildo Rodrigo Disner, Carla Lima

**Affiliations:** 1 Immunoregulation Unit, Laboratory of Applied Toxinology (CAT/CeTICS-FAPESP), Butantan Institute, São Paulo, SP, Brazil.; 2 Graduate Program in Environmental Sciences, Federal University of Tocantins, Palmas, TO, Brazil.; 3 Graduate Program in Systematics, Use, and Conservation of Biodiversity, Department of Biology, Center for Sciences, Federal University of Ceará, Fortaleza, CE, Brazil.; 4 Department of Dermatology, Botucatu Medical School (FMB), São Paulo State University (UNESP), Botucatu, SP, Brazil.

**Keywords:** Brazilian venomous fish, Biochemical activities, Toxic effects *in vivo*, Comparative study, Envenomation

## Abstract

**Background:**

Brazilian waters are home to various venomous fish species, each with its unique venom composition. Although common, envenomation cases are largely underreported, leading to a lack of public health policies for prevention and treatment. Some of the most clinically relevant fish in Brazil include the stingray *Potamotrygon orbignyi*, the toadfish *Thalassophryne nattereri*, the scorpionfish *Scorpaena plumieri*, and the catfish *Pseudoplatystoma fasciatum* and *Cathorops spixii*.

**Methods:**

We comprehensively searched reports about accidents involving venomous fish in Brazil and compared the toxic activities of some medically relevant species.

**Results:**

From the biochemical and toxicological evaluation, we found that venoms show a hierarchy in the ability to induce local toxic effects in mice, probably related to the venom compound diversity with species-specific toxins. *T. nattereri* venom presents greater toxicity, causing more severe local responses than that of *P. orbignyi*, *C. spixii*, and *P. fasciatum*, which cause moderate reactions. The *S. plumieri* venom induced only a moderate level of edema and could not cause nociception or necrosis. These results highlight that envenomation by *P. orbigny*, *C. spixii*, and *S. plumieri* is marked by proteins with intense hemolytic/proteolytic and phospholipase activity. On the other hand, *T. nattereri* and *P. fasciatum* offered a broader panel of new toxin families.

**Conclusion:**

Knowledge of fish venom biochemical and toxicological activities is crucial to antivenom therapy development and helps endorse the study of venomous fish and their impact on the public health system.

## Visual Abstract

**Figure:**
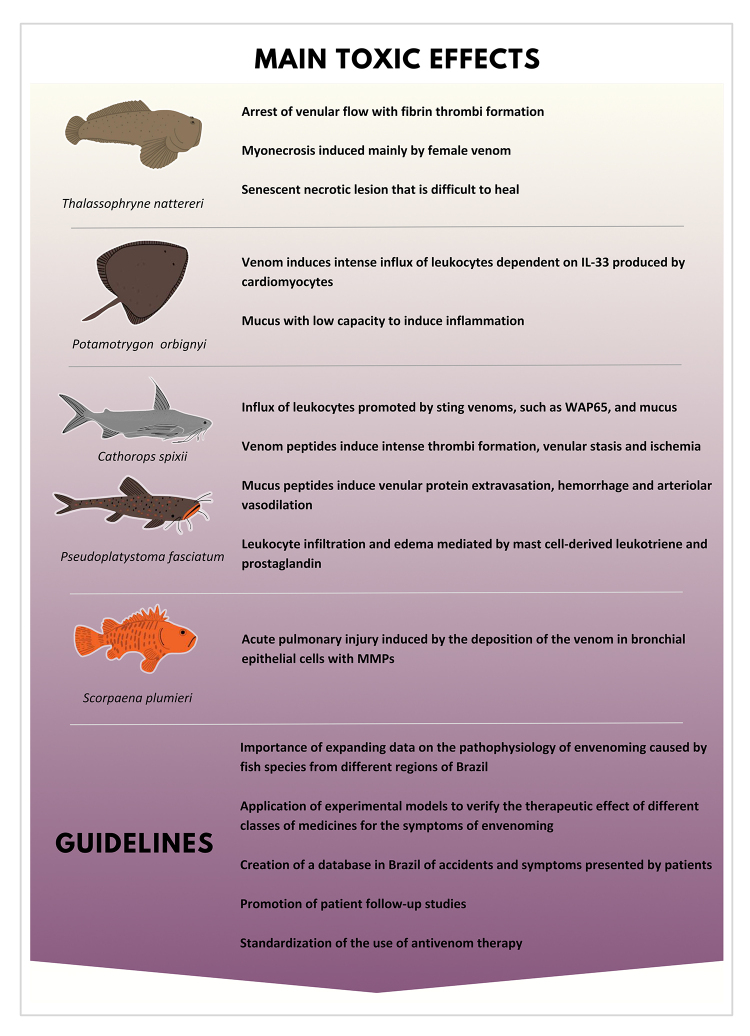
Main Toxic Effects

## Background

The Brazilian territory is one of the most water-abundant globally, characterized by a diverse range of aquatic ecosystems encompassing extensive marine and continental systems. Brazil is estimated to hold 12% of all surface freshwater, divided into 12 hydrographic regions and numerous micro-basins. The marine environment is encompassed by one of the longest coastlines in the world, with more than 8,698 km in length and 12 nautical miles of the territorial sea [1-3]. Such a rich aquatic environment has perfectly served as a habitat for countless fish species and other aquatic life. Brazil’s temperate and tropical waters are home to virtually all families of the nearly 200 species of fish considered venomous or poisonous. The country’s most popular venomous species are toadfish (popularly known as “*niquim*”), catfish, scorpionfish, and stingray. 

The prevalence of venomous fish in Brazil has led to increasing attention on related accidents, primarily due to the significant morbidity associated with the injuries despite their non-fatal nature. In contrast to terrestrial venomous animals, the distinctive chemical composition of fish venoms [4, 5] is responsible for unconventional clinical manifestations characterized mainly by swollen, painful, and difficult-to-heal necrotic lesions commonly found in accidents caused by *niquim*, catfish, and stingrays [6]. Systemic effects include cardiotoxicity, hypotension for scorpionfish [7], and anaphylaxis caused by *T. nattereri* natterin [8]. Notably, local lesions are difficult to treat with medications commonly used to control inflammation-mediated pain and swelling. As a result, victims experience painful lesions that can last from days to months, eventually resulting in necrosis.

Various unconventional and ineffective methods have been proposed to alleviate symptoms, including hot water or 5% vinegar immersion and saline rinses. However, no specific therapy has been established to neutralize the effects of the toxins [9], except for the polyspecific stonefish antivenom used in Australia for incidents caused by Scorpaenidae fish species such as *Synanceia horrida* and *Synanceia verrucosa* [10]. The lack of adequate therapy for fish envenoming highlights the urgency of further research on managing accidents, monitoring clinical complications, identifying the toxins responsible for pathophysiology, and the type of specific drug class to control the symptoms. 

Despite the considerable occurrence of fish envenoming incidents in different Brazilian regions, which represent a public health problem, these cases are significantly neglected [11]. The lack of compulsory notification for fish-related accidents, especially in underdeveloped areas, contributes to this underreporting. As a result, there is a substantial gap in the scientific literature and understanding of aquatic venomous animals’ epidemiology. This lack of data hampers the ability of researchers and public health professionals to fully comprehend the problem and design effective prevention and treatment strategies [6].

Therefore, the objective of this study was to collect and compile data from the literature and case reports concerning accidents involving venomous fish in Brazil over the past decade and to investigate the biochemical properties and primary toxic activities of the venoms from some clinically relevant species. Altogether, these endeavors might support a deeper understanding of the venom’s specific toxic features and help to promote the development of particular treatments.

## Methods

### Bibliographic survey

The bibliographic survey was conducted by a comprehensive search in databases for studies reporting accidents caused by venomous fish. Articles, communications, conference proceedings, theses, and dissertations were considered to expand the search spectrum as much as possible.

Additionally, at the beginning of this review, we explored all the most common databases, *i.e.*, Scopus, Scielo, Web of Science, PubMed, and Google Scholar. We realized that PubMed and Google Scholar provided the most extensive range of publications of interest, notably covering Central and South American countries, where the risk of envenoming by fish is higher. The other databases either provided a low number of publications or were mainly represented by out-of-the-scope publications. 

The search term “venomous fish in Brazil” was used on each selected platform, and the inclusion criteria considered studies published in Portuguese or English in the last ten years, *i.e*., from January 2013 to October 2023. Results referring to reviews and books, studies with poisonous animals besides fish, toxins research not exactly in envenomation accidents, and incidents due to fish toxins ingestion were disregarded.

### Fish and venoms

**Figure 1.**
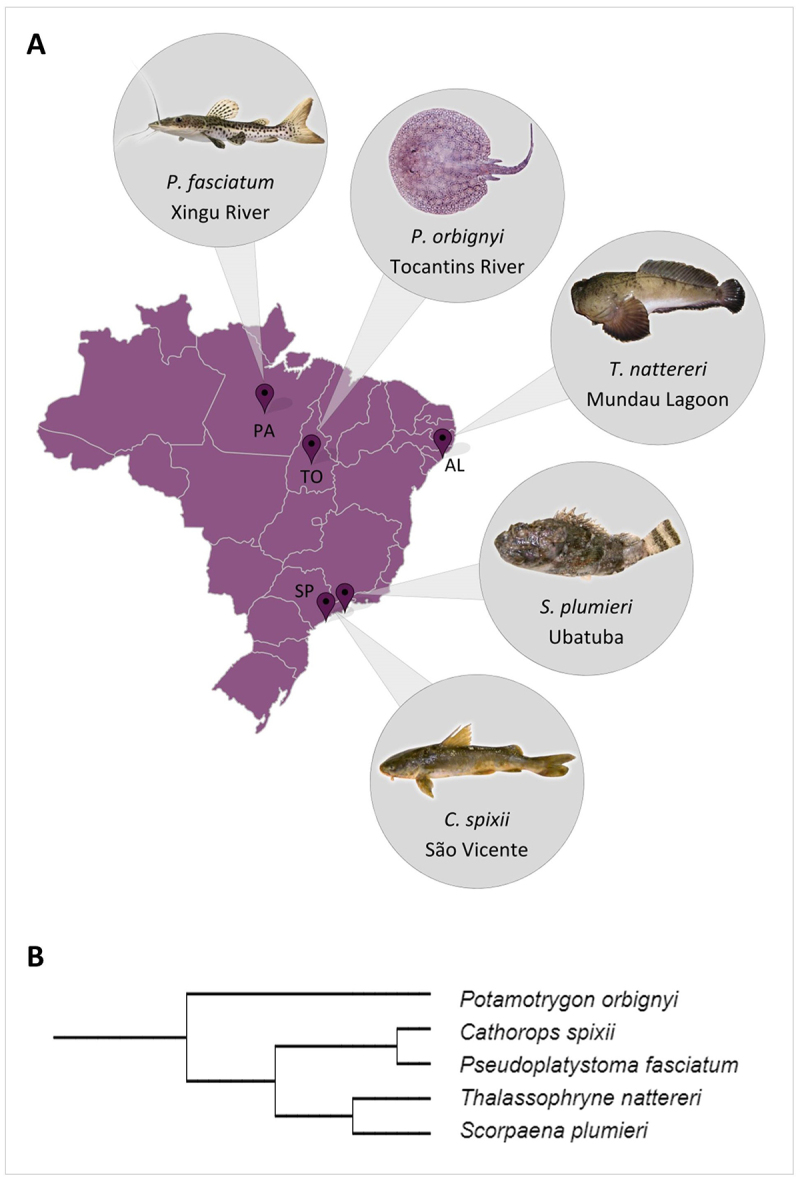
Geographic location of the main species of venomous fish in Brazil used in this study. **(A)** Adult specimens of the toadfish *Thalassophryne nattereri*, the scorpionfish *Scorpaena plumieri*, the catfish *Cathorops spixii* were collected on the coast of the state of Alagoas (Mundau Lagoon) and São Paulo (Ubatuba and São Vicente cities) and the continental stingray *Potamotrygon orbignyi* and the catfish *Pseudoplatystoma fasciatum* in the rivers of the state of Tocantins (Tocantins and Xingu Rivers), respectively. **(B)** Phylogenetic tree illustrating the evolutionary connections between Brazilian venomous fish species studied.

Adult specimens of the stingray *Potamotrygon orbignyi*, toadfish *Thalassophryne nattereri*, scorpionfish *Scorpaena plumieri,* catfish *Pseudoplatystoma fasciatum,* and *Cathorops spixii* were collected in the Brazilian states of Alagoas, Tocantins, Xingu River at Para State and São Paulo (Figure 1 A ). Animal collection was authorized by the Brazilian Institute of Environment and Renewable Natural Resources (IBAMA - *Instituto Brasileiro do Meio Ambiente e dos Recursos Naturais Renováveis*), under processes no. 14693-1 and no. 45407-1. Right after capture, the extraction of the venom of *T. nattereri* was carried out by compressing the base of the spines (each lateral and two dorsal), forcing the expulsion of the venom [12]; for collection of *S. plumieri* venom, the integumentary sheath covering each spine was stripped to the base of the spine and the venom was aspirated from the glandular grooves with a micropipette [13]; for *C. spixii*, *P. fasciatum* and *P. orbignyi*, the collection was through maceration in PBS pH 7.4 of the glands that cover the spines (each lateral and one dorsal) [14-16]. The recovered fish or their stingers were kept in boxes with ice during collection and transport to the laboratory. If the stingers were shipped, they were shipped frozen in nitrogen cylinders. In the laboratory, after processing (4,000 rpm, 15 min., 4 °C), the venoms were aliquoted into 20 uL minieppendorfs and stored at -20 °C. We avoided manipulating the samples and minimized freezing and thawing cycles as much as possible to maintain the stability of the venom. Stored venoms were tested for toxic activities by intravital microscopy, as a standard procedure before carrying out experiments.

### Phylogenetic analysis

The phylogenetic tree was generated using the software PhyloT v2 (https://phylot.biobyte.de/, accessed on 01/Feb/2023) and displayed by the Interactive Tree Of Life (iTOL) system (https://itol.embl.de/, accessed on 01/Feb/2023) according to Letunic & Bork [17]. PhyloT generates phylogenetic trees based on the NCBI taxonomy or Genome Taxonomy Database (GTD).

### Mice

Swiss male mice (7-8 weeks old; 18-22 g) were obtained from a colony at Butantan Institute. Mice were maintained in sterile microisolators with sterile rodent feed and acidified water and housed in positive-pressure air-conditioned units (25 °C, 50% relative humidity) on a 12 h light/dark cycle. All experiments were performed under the National Council for Animal Experiment Control (CONCEA) laws and approved by the Butantan Institute's Animal Use Ethics Commission (#379/07).

### Protein determination and sodium dodecyl sulphate-polyacrylamide gel electrophoresis (SDS-PAGE)

The protein concentration in the venom extracts was determined by Bradford’s colorimetric method [18] using bovine serum albumin (Sigma, St. Louis, MO, USA) as the standard. The amount of venom was expressed by its protein content in mg/mL. The endotoxin content was also evaluated to ensure that the venom extracts were free from significant contamination that could interfere with the study’s results. Endotoxin content was measured using the QCL-1000 chromogenic Limulus amoebocyte lysate assay (Bio-Whittaker) according to the manufacturer’s instructions, confirming that samples contained less than 0.8 pg LPS. The electrophoretic profile of venoms was evaluated using 10 (g of each venom in a 4-20% SDS-PAGE acrylamide gradient under non-reducing conditions, stained with silver using the method of Laemmli [19]. Protein Ladder (18-135-100-75-48-35-25-17-11 kDa) #SP007-0500, Universal Biotech was used for molecular weight estimation. Protein bands were visualized by Coomassie R-250.

### Toxic induced activities

Each mouse (*n* = 5/group) was kept in an adapted chamber mounted on the mirror for nociceptive tests. After a 10-minute adaptation period, mice received intraplantar injection of venoms (30 (g of each venom/animal) into the right footpad in a fixed volume of PBS (30 (L). The control group was injected only with sterile PBS. Then, the mice were returned to the observation chamber, and the amount of time spent licking or biting each hind paw was recorded for 30 min and taken as the index of nociception [15]. Likewise, independent groups of mice were used for edema evaluation (*n* = 5) following the same exposure method. Local edema was quantified by measuring the thickness of injected paws with a pachymeter (Mytutoyo) after 2 h of venom injection. The results were presented by the difference between experimental and control footpad thickness [14].

For necrosis evaluation, 50 µg of each venom diluted in 50 µL of PBS was intradermally injected into the shaved backs of mice. After 72 h, they were euthanized, and the skin was removed to measure the necrotic area. Two diameters were determined for the necrotic spot: the longest and its perpendicular one [20].

### Determination of hemolytic, proteolytic, and phospholipase activities

Human erythrocytes from a healthy donor (type A) were collected in 0.15 M citrate buffer, pH 7.4, and washed 3 times by centrifugation with 0.15 M phosphate-buffered saline, pH 7.4. To determine the hemolytic activity, 50 µg of each venom in 100 µL was added to a solution of 3% erythrocytes in wells of U-shaped bottom plates and incubated for 3 h at room temperature. A solution of 3% erythrocytes incubated with water or PBS was considered positive or negative control, respectively [21]. Assays were run in triplicate including appropriate blanks. After incubation, the U-plates were centrifuged at 1700× *g* for 5 min, and then 100 μL of the supernatants were transferred to transparent, flat-bottom 96-well plates. Finally, absorption was measured at 595 nm in spectrophotometer (Titertek Multiskan, EFLAB, Finland) and the percentage of hemolysis in relation to the positive control was calculated. This procedure is according to Sæbø *et al*. [22] and Carducci *et al*. [23].

The proteolytic activity was estimated using an N,-N-dimethylated casein (Sigma) as a substrate in a system containing 400 μL of buffer solution (0.1 M Tris-HCl buffer, pH 8.8, 0.01 M CaCl2), 100 µL contained 30 µg of each of venom and 500 µL of 2% casein solution (solubilized in the same buffer), for 30 min, at 37 °C. The reaction was stopped by adding 1000 μL of 5% trichloroacetic acid (TCA). After incubation, the 3 mL tubes were centrifuged at 1700× *g* for 5 min, and then 100 μL of the supernatants were transferred to transparent, flat-bottom 96-well plates. Finally, absorption was measured at 280 nm in spectrophotometer (Titertek Multiskan, EFLAB, Finland). This procedure is according to Herrera *et al*. [24] and Lin *et al.* [25]. Assays were run in triplicate including appropriate blanks, which were prepared by combining the TCA with the enzyme and then adding the casein substrate. Enzyme units per mL (U/mL) and enzyme units per mg (U/mg) were calculated as follows: U/mL = ((AS280-AB280) x 2 x DF) / 0.5 and U/mg protein = (U/mL) / (mg protein/mL enzyme); where AS280 and AB280 are absorbance values of sample and blank, respectively; 2, total volume (mL) of assay; DF, sample dilution factor; and 0.5, volume (mL) of enzymatic extract.

The phospholipase activity assay used 4-nitro-3-(octanoyloxy) benzoic acid (4N3OBA) as a colorimetric reagent. 4N3OBA (Sigma, Castle Hill, New South Wales, Australia) was dissolved in chloroform at a concentration of 50 mg/mL. Aliquots (80 μL, 4 mg) were distributed into Eppendorf tubes and evaporated under vacuum. The dry 4N3OBA residue (4 mg per tube) was stored at −20 °C. Immediately before the assay, substrate (4 mg) was resuspended in 1 mL of PLA_2_ assay buffer (150 mM KCl, 10 mM CaCl_2_, 50 mM Tris-HCl, pH 7.5). The suspension was vortexed vigorously for 1 min and centrifuged (2,000 *g*, 2 min) at room temperature, and the supernatant was used as a substrate solution (190 μL) in enzyme assays. In a 96-well plate, 10 μL contained 30 µg of each of venom and 190 μL of 4N3OBA solution, in triplicate, were dispensed. Distilled water was used as the negative control. The plate was incubated in a spectrophotometer at 37 °C, and the optical density (OD) was measured at 425 nm. The activity was expressed as a unit of chromophore released/mg protein referring to the amount of enzyme required to hydrolyze a specific quantity of substrate at 30 min according to de Melo *et al*. [26] and Petrovic *et al*. [27].

### Evaluation of microcirculatory alterations in cremaster muscle

Mice (*n* = 5/group) were anesthetized by an intraperitoneal injection of 2% xylazine (Calmiun^®^, Agener União, SP, Brazil) and with 0.5 g/kg of ketamine (Holliday-Scott SA, Buenos Aires, Argentina). The scrotum was opened, and the cremaster muscle exteriorized. After longitudinal incision with cautery and spreading of the muscle over a cover glass, the epididymis and testis were mobilized and pinned aside, leading to full microscopic access to the cremaster muscle microcirculation. Cremaster muscle of mice was exposed to a topic application of 20 (L of 10 (g of venom of *C. spixii, P. fasciatum, P. orbignyi, S. plumieri*, and *T. nattereri* in sterile PBS. Negative-control mice were submitted to the topic application of sterile PBS. The exposed tissue was superfused with 37 °C warmed bicarbonate-buffered PBS, pH 7.4. The post-capillary venules with a diameter of 25-40 μm were chosen using the software associated with intravital microscope Axio Imager A.1 (Axiolab, Carl Zeiss), and the interaction of leukocytes with the luminal surface of the venular endothelium was evaluated by counting the number of rolling leukocytes every 10 min after application of each venom for 30 min. Rolling leukocytes were defined as those moving at a velocity less than erythrocytes (reduced to approx. 5 μm/s) and demonstrated a clear rolling motion. Intravital microscopy was conducted for 40 min on an upright microscope (Axiolab, Carl Zeiss, Oberkochen, Germany) with a saline immersion objective (SW40/0.75 numerical aperture, Zeiss, Jena, Germany) coupled to a photographic camera (AxioCam Icc1, Carl Zeiss, Oberkochen, Germany) using a 10/0.3 longitudinal distance objective/numerical aperture and 40x or 100x and 1.6 optovar (Carl Zeiss, Oberkochen, Germany) according to dos Santos *et al*. [28].

### Zymography

To visualize gelatinolytic enzymatic activity in the venoms, electrophoresis (SDS-PAGE) was performed using gels of 10% polyacrylamide co-polymerized with 1 mg/mL gelatin (EC61752BOX, Invitrogen) and venoms at 20 μg each/well. Gels were washed for 30 min in a buffer containing 50 mM Tris-HCl (pH 7.5), 5 mM CaCl_2_ (Sigma), and 2,5% Triton X-100 and incubated overnight (16 h) in incubation buffer at 37 °C (50 mM Tris-HCl, 5 mM CaCl_2_, 0.02% NaN_3_), pH 7.6 (Sigma) and 1% Triton X-100. Gels were stained with Coomassie blue and discolored by acetic acid in methanol and H_2_O (1:3:6) for 60 min. In this method, the proteolytic activity on gelatin is detected as colorless bands on the otherwise blue gel after staining with Coomassie blue [13]. For Zymogran gel was used the Prestained Protein Marker (180-140-100-75-60-45-35-25-20 kDa), #PL00001, Proteintech.

### Statistical analysis

All values were expressed as the mean ± SEM of three independent experiments using *n* = 5 mice/group. Parametric data were evaluated using analysis of variance, followed by the Bonferroni test for multiple comparisons. Non-parametric data were assessed using the Mann-Whitney test. Differences were considered statistically significant at *p* < 0.05. The GraphPad Prism 6 (Graph Pad Software, v6.02, 2013) statistical package was employed.

## Results and discussion

### Publications on accidents with venomous fish in Brazil from 2013 to 2023

**Figure 2.**
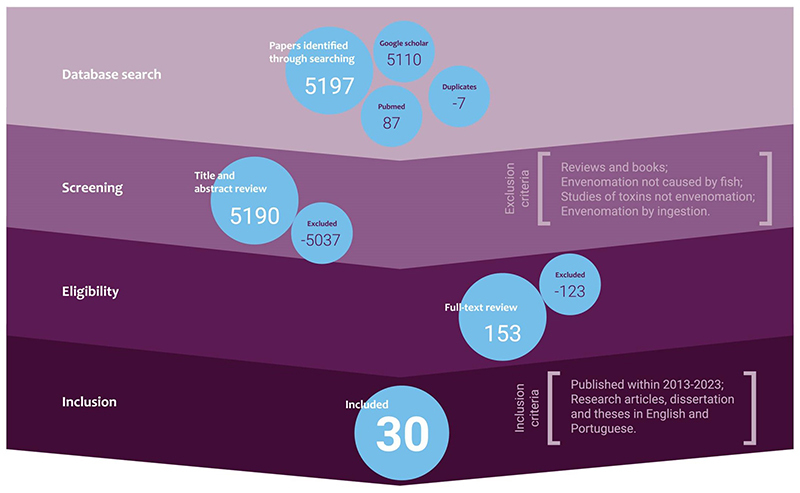
Screening process used to select articles with greater relevance to accidents involving venomous fish in Brazil from 2013 to 2023. The search term “venomous fish in Brazil” was applied on PubMed and Google Scholar following determined inclusion and exclusion criteria.

The initial database search on accidents with venomous fish in Brazil resulted in 5,197 titles, combining the two platforms (*i.e.*, PubMed and Google Scholar). Three steps were carried out to reach the final eligible publications. In the first stage of evaluation, seven duplicates were excluded. In the second stage, the contents of the titles and abstracts were evaluated, where 5,037 works were excluded. In the third stage, a thorough analysis was carried out to cover the entire document; in this process, 123 publications were further excluded. Finally, 30 publications met all the requirements (*i.e*., inclusion and exclusion criteria) and were included in the study. This result comprised 24 published articles, three dissertations, two conference proceedings, and a monograph (Figure 2).

**Figure 3.**
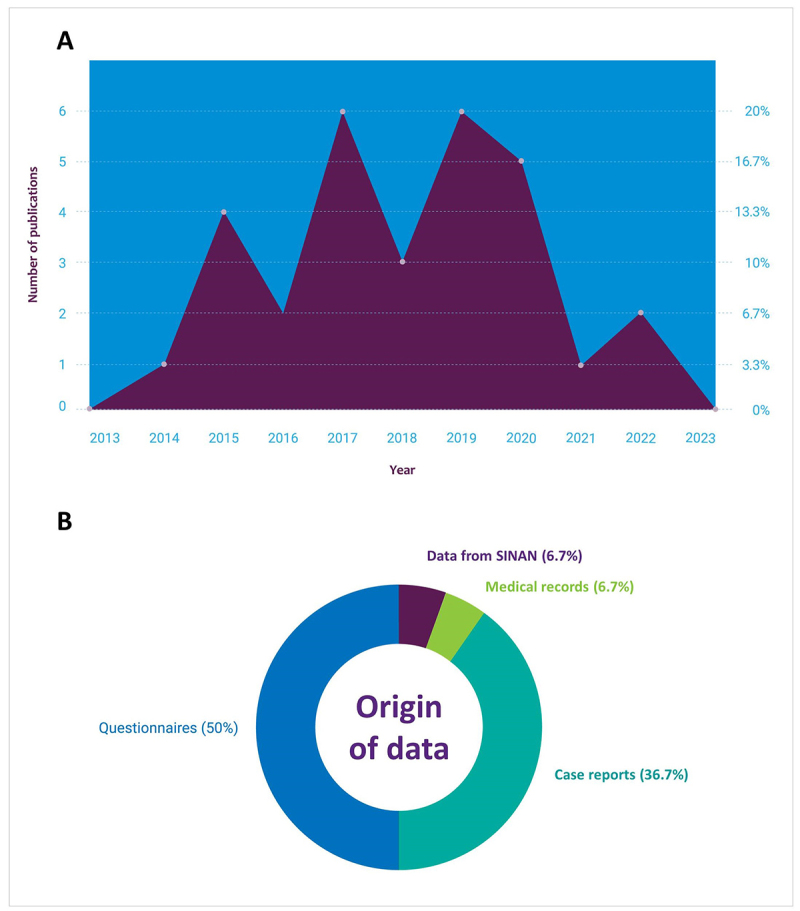
Trends and data sources in research on venomous fish accidents in Brazil. **(A)** Number of publications per year in the last ten years about accidents involving venomous fish in Brazil. **(B)** Origin of the data included in the reviewed studies. Note: SINAN stands for “*Sistema de Informação de Agravos de Notificação*”, a national system powered mainly by the notification and investigation of cases of diseases and injuries that appear on the national list of notifiable diseases.

Throughout the evaluated period, numerous studies were published, with the highest number of publications occurring in the years 2017, 2019, and 2020. The years 2017 and 2019 had six publications recorded each (20.0%), followed by 2020, with five publications (16.7%) (Figure 3 A).

The data collected by the publications presented different collection methods, the most common being the application of questionnaires/interviews totaling 15 documents using this method (50%); 11 case reports (33.3%); two data recorded/compiled through the Notifiable Diseases Information System - SINAN (6.7%) and two evaluations of medical records (3.3%) (Figure 3 B ). Reckziegel *et al*. [11] and Sachett *et al*. [29] had already reported the difficulty of obtaining information through the Notifiable Diseases Information System, the possible failures, and associated concerns. Reckziegel *et al*. [11] reviewed data on injuries caused by aquatic animals in Brazil from 2007 to 2013 using the SINAN.

**Figure 4.**
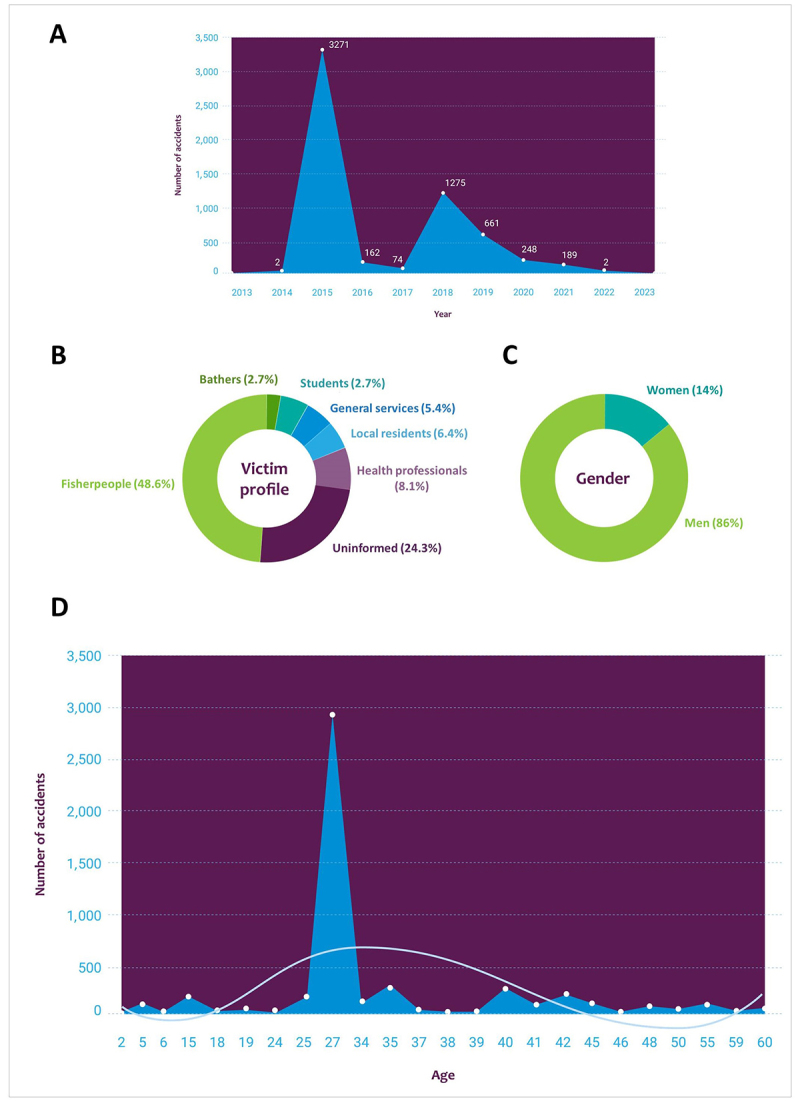
Characteristics of venomous fish accident victims in Brazil over the last decade. **(A)** Number of accidents per year reviewed in the last ten years’ publications. **(B)** Percentage of accidents by victim profile, classified as fisher people, health professionals, local residents, general services, bathers, students, and not informed. **(C)** Gender of people who suffered accidents with venomous fish in the studies analyzed over the last ten years. **(D)** Age average of the venomous fish victims reported in the reviewed papers. Papers where the age was not mentioned, or the range was too large were not included. The tendency line evidences the age distribution of the reported cases with the polynomial distribution determining the fluctuations in the data.

Temporal analysis reveals fluctuating incidents, with peaks in 2015 and 2018 and troughs in 2014 and 2022 (Figure 4 A ). These variations could be influenced by multiple factors, including underreporting by the healthcare system and individuals opting for self-treatment instead of seeking medical attention. Challenges in obtaining accurate data from the health information system, as noted, further complicated efforts to assess the true extent of venomous fish envenomation in Brazil.

Seeking to better understand the primary victims of accidents involving venomous fish in Brazil, we analyzed the victims’ profiles, including gender and age. Thirty-six profiles of fish victims were identified. People having fishing as their occupation are the group most affected by accidents involving venomous fish, corresponding to 48.6% of cases in the last ten years, followed by “health professionals” with 8.1% of reported cases. The other categories, “residents”, “general services” and “students” presented the same percentages, accounting for 5.4% of reports, while “bathers” presented the lowest value with only 2.7% of cases. It is worth noting that 24.3% of the documents did not mention or provide information about the profile of the victims, which are categorized as “Not Informed” (Figure 4 B ). Of these, the most affected gender was male, with 86% corresponding to 4,821 accidents, compared to females, which presented a percentage of 14%, equivalent to 785 accidents in the last ten years (Figure 4 C ). Only one study did not present data regarding gender. The profile obtained in our survey corroborates several other studies [29, 30, 31]. 

The predominance of male victims in these types of accidents is consistent with other reports, likely due to the higher engagement of men in activities close to stingrays, such as fishing. Another noteworthy aspect of such incidents is the reliance on diverse and sometimes unconventional treatment methods, including traditional remedies. This pattern is commonly observed across various geographic regions where fish injuries are prevalent, highlighting cultural and practical responses to these accidents [32].

Health education is also deficient for health professionals and the fishing population, crucial to improving awareness and response to fish envenomation. Haddad *et al*. [33] state that in a study of over 400 stingray injuries in Brazil, the majority occurred in fishermen, corroborated by Aquino *et al*. [34], who stated that injuries caused by venomous fishes are frequent and important in Brazilian freshwater environments.

Regarding the age of the victims, both the absolute ages reported in the studies were considered, as well as the averages calculated for those who only presented age ranges without an absolute value, and subsequently related to the numbers of accidents. In total, 22 articles contained sufficient information about the age of the injured individuals; those that did not include such details were considered within the “Not Specified” class. As depicted in Figure 4 D , accidents occur in practically all age groups, from children to older people. However, the most affected group is between 20 and 40, with a prevalence of around 27 years.

**Figure 5.**
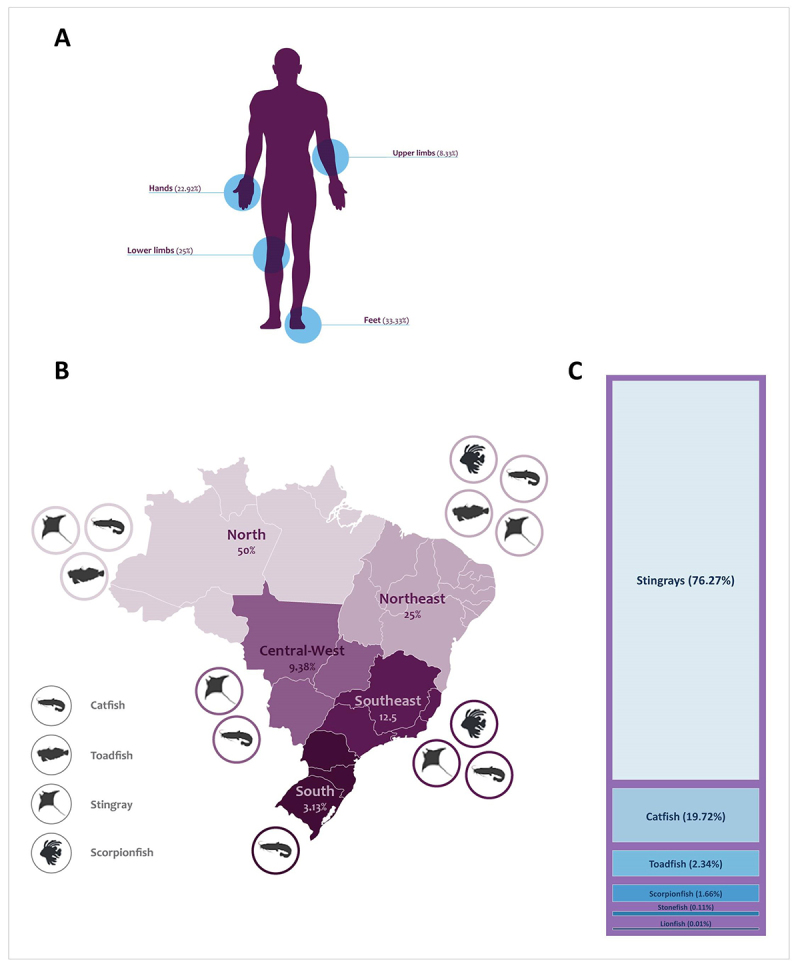
Body regions, geographic distribution, and species involved in venomous fish accidents in Brazil. **(A)**The regions of the body most affected in accidents with venomous fish registered in the reviewed studies. The other body parts not illustrated represent 7.9% of the reports; still 2% did not specify the affected area. **(B)** Representation of the distribution by region of the number of accidents involving venomous fish in Brazil according to the articles analyzed and the prevalent species. **(C)** Percentage of the most reported fish species that caused the accidents in the reviewed papers. The percentage was obtained by counting accidents by species in each paper.

Another element assessed was the victims’ body parts most affected during accidents based on the 30 studies screened (Figure 5 A ). Of these studies, the majority indicated feet (33.33%) as the most affected area, followed by hands (22.92%) and lower limbs (25%), such as calves and thighs. Other regions, such as the head, neck, and chest, were reported in smaller proportions.

These injury patterns are particularly concerning during low water and dry seasons when stingrays concentrate in shallow areas where fishermen often work. This proximity increases the likelihood of incidental catches and, unfortunately, injuries when fishermen accidentally step on or mishandle them [33, 35, 36].

Encounters with venomous fish leading to injuries are becoming increasingly prevalent, particularly in Brazil’s northern and northeastern regions (Figure 5 B ). This phenomenon can be attributed to several factors, including the significant role of these areas in artisanal fishing and the emergence of beachgoers during warmer seasons and school vacations. The interaction between humans and venomous fish is more frequent in these areas due to walking barefoot on beaches and handling fish without adequate protection, leading to a higher incidence of contact and subsequent injuries. Additionally, the distribution patterns of certain species, such as the *niquim*, appear to contribute to the concentration of incidents in these regions.

Analysis of data collected over the past decade reveals a concerning trend, with stingrays emerging as the primary cause of fish-related accidents, accounting for 76% of reported cases (Figure 5 C ). This finding is consistent with previous research [11], which documented many stingray-related injuries in the northern region between 2007 and 2013. Additionally, there is a notable incidence of catfish accidents, comprising 20% of reported cases during the same period. Interestingly, while stingrays dominate the statistics nationally, studies like Carvalho *et al*. [37] highlight variations in regional patterns, such as a higher prevalence of catfish-related incidents in Maranhão state. 

### Venoms show a hierarchy in the ability to induce local toxic effects in mice

Our epidemiological data show that stingrays, catfish, toadfish, and scorpionfish are the species most frequently involved in accidents with humans in Brazil. So, our next step was to collect these species to study their venoms comparatively. Adult specimens were used to obtain enough crude venoms to determine their relative position on a hierarchical toxicity scale, comparing the main characteristics of the envenomation in a murine system.

Large-scale duplication events are likely the underlying mechanism responsible for the adaptive changes that gave the fish in this study the ability to adapt to different environments and diversify their tools of defense and attack, including their venom apparatus [38, 39]. The evolutionary connections among these species can be observed, including monophyletic groups between the Batrachoidae and Scorpaenidae family representatives (*T. nattereri* and *S. plumieri*) and between the two catfish from the Ariidae family (*C. spixii* and *P. fasciatum*), and stingray in a separated branch of the tree (Figure 1 B ). The toadfish has the most developed venomous apparatus with the two hollow spines of the dorsal fin and each hollow lateral spine attached to glands that conduct the venom through the canal, unlike the scorpionfish with glands distributed in grooves along each side of the 13 dorsal spines, two pelvic and three anal spines. Additional file 1 provides detailed information on phylogenetic classification and biological aspects of the covered species.

Catfish are some of the most abundant venomous fish in Brazil. Small catfish species like *C. spixii* present long and robust saw-tooth-shaped spines, one on the dorsal fin and one on each pectoral fin. *Pseudoplatystoma* is an economically important genus of pimelodids from South America, with large species and strong spines, and both catfish have venom glands distributed along serrated spines. In contrast, the potamotrygonid stingrays are the only group of elasmobranchs to have evolved exclusively within freshwaters environments, with envenomation caused by toxins produced by glands dispersed along the stingers, which are bilaterally positioned in the whip-like caudal appendage (Additional file 1).

**Figure 6.**
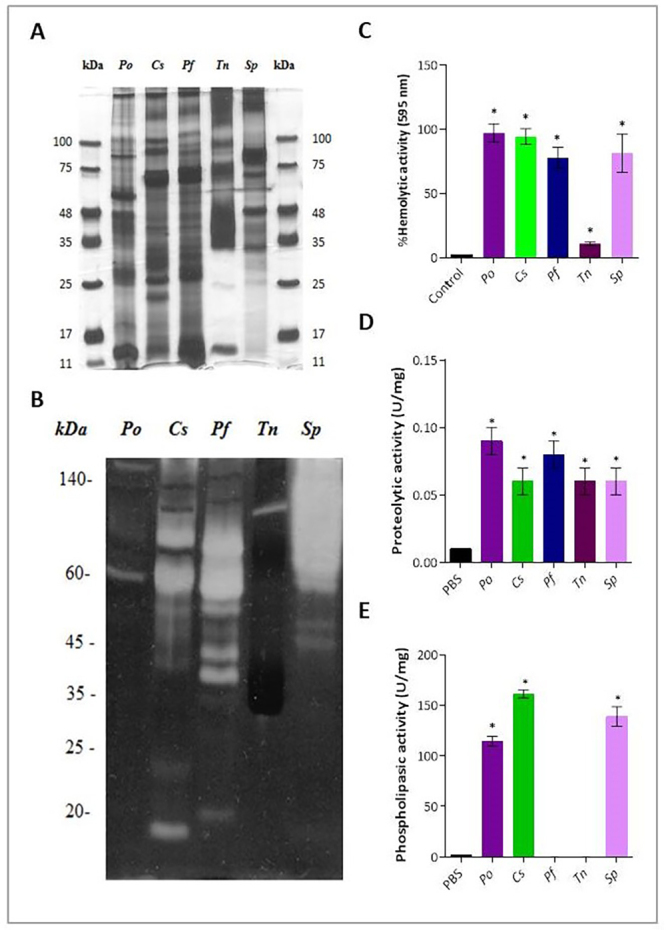
Comparison of the hemolytic, proteolytic, and phospholipasic activities of venoms. **(A)** The electrophoretic profile of venoms was evaluated using 10 (g of each venom in a 4-20% SDS-PAGE acrylamide gradient under non-reducing conditions, which was stained with silver. The numbers on the left and right correspond to the position of the MW markers. (Po) *Potamotrygon orbignyi*, (Cs) *Cathorops spixii*, (Pf) *Pseudoplatystoma fasciatum*, (Tn) *Thalassophryne nattereri*, (Sp) *Scorpaena plumieri*. **(B)** Zymographic gelatin of venoms. Gels of 10% polyacrylamide co-polymerized with 1 mg/mL gelatin were run with venom at 20 (g each/well. The numbers on the left correspond to the position of the MW markers. **(C)** Hemolytic activity, 3% human erythrocytes were incubated with 50 µg of venoms in 100 µL for 3 h at room temperature. Hemolysis was determined by reading the absorbance at 595 nm. **(D)** Proteolytic activity, N,-N-dimethylated casein was used as a substrate in a buffer solution containing 30 μg of venoms, for 30 min, at 37 °C that was evaluated at 280 nm. **(E)** Phospholipase activity assay, 4-nitro-3-(octanoyloxy)benzoic acid (4N3OBA) was used as a colorimetric reagent with 10 μL of venoms in triplicate and evaluated at 425 nm with kinetic intervals of 30 s over a period of 30 min. *p < 0.05 compared to the negative control group.

The structural and pharmacological diversity of fish venom components may be related to the hyper-diversification of the group [40]. Selection pressures in the aquatic environment may have driven each species to complex adaptations, such as the composition of venoms and their role as toxins. The diversity of toxins in fish venoms contrasts with the typical patterns of functional evolution of other toxin gene families from the venoms of sea anemones, cone snails, spiders, scorpions, centipedes, and snakes [41]. Next, we performed a comparative analysis of the toxic activities of selected Brazilian fish venoms. In Figure 6 A , we confirmed in SDS-PAGE gel the electrophoretic profiles of the venoms described elsewhere, including high-intensity bands around 11-17 kDa in the sting venoms of *P. orbignyi*, *C. spixii*, *P. fasciatum*, and *T. nattereri*; around 25 kDa in the venoms of *P. orbignyi*, *C. spixii*, and *P. fasciatum*. Furthermore, high-intensity bands around 35-48 kDa were observed in the venoms of *P. orbignyi*, *T. nattereri*, and *S. plumieri*. Finally, protein bands with elevated molecular weight above 48, around 75 and above 100 kDa were detectable in all venoms.

Then, venom proteins with proteolytic activity that may be involved in the pathophysiology of envenomation were compared (Figure 6 B ) and we found that both catfish venoms (*C. spixii* and *P. fasciatum*) have almost similar gelatin hydrolysis profiles mainly around and above 60 kDa. In addition, *C. spixii* venom showed gelatinolytic activity below 20 kDa, and the venom of *P. fasciatum* presented two unique high-intensity bands between 35-45 kDa. *P. orbignyi* and *T. natereri* venoms showed only one low-intensity gelatinolytic band around 60 or 100 kDa, respectively. High intensity gelatinolytic bands with molecular mass above 60 kDa were seen in the *S. plumieri* venom.

The mechanism of action of hemolytic toxins is unclear, but binding to sialic acid present in the glycocalyx, glycoproteins, or glycosphingolipids on the membrane surface has been proposed, followed by membrane rupture [42]. In Figure 6 C , we observed that except for the *T. nattereri* venom, all venoms exhibited high hemolytic activity against human erythrocytes (97% ± 7 for *P. orbignyi*; 94% ± 6 for *C. spixii*, 78% ± 8 for *P. fasciatum*, and 81% ± 15 for *S. plumieri* compared to negative control; p < 0.05). Regarding proteolytic activity on casein as a substrate, all venoms showed this ability (Figure 6 D ), but only the venoms of *P. orbignyi*, *C. spixii*, and *S. plumieri* showed phospholipase activity (Figure 6 E ). Previous studies have revealed that the dimeric cytolytic protein Sp-CTx (80 kDa) involved in the local effects of scorpionfish may be accompanied by a hypertensive response and bradycardia [7]. In contrast, the senescent injury induced by the toadfish [43], accompanied by systemic manifestations such as pulmonary neutrophilic inflammation and anaphylaxis, is caused by natterin proteins [8, 44, 45]. Hyaluronidases have been found in many stingray venoms, including the freshwater *Potamotrygon* sp. and marine *Dasyatis guttata* [46-48].

Our comparative results show that all venoms in this study, especially from *C. spixii*, *P. fasciatum*, and *S. plumieri* are composed of different proteinases, including gelatinases and phospholipases, which may be responsible for some pathological activities triggered by these venoms. Protein toxins in *P. orbignyi* venom remain to be identified, as so far only peptides have been characterized such as Orpotrin effective in microcirculatory environment causing a strong vasoconstriction [49]. In addition, the Wap65-like protein (warm temperature acclimation-related protein 65 kDa) has been described in *C. spixii* venom [50].

Borges *et al*. [51] used mass spectrometry to analyze toxins from the venom of *Scorpaena plumieri* collected on shallow water beaches on the coast of Espírito Santo state, Brazil, and were unable to identify proteins homology with phospholipase (PLA). On the contrary, recently Tang *et al.* [52] demonstrated that in addition to the toxins already described as metalloproteinases as found by Ziegman *et al*. [53], C-type lectins by Andrich *et al*. [54], stonustoxin [SNTX] by Low *et al*. [55] and Poh *et al*. [56], reef stonefish as well as other toxic species *Scorpaenoidei* fishes also present another 103 toxins, including natterin [57], phospholipase, dipeptidyl peptidase, hyaluronidase, serine protease, phosphodiesterase, cystatin, coagulation factor, acetylcholinesterase, cysteine-rich venom protein, cytolysin, Kunitz-type protease inhibitor, thalatoxin, and other 37 families with only one toxin gene in each family. These results highlight that the envenomation induced by the venoms of *P. orbigny*, *C. spixii*, and *S. plumieri* can be attributed to proteins with intense hemolytic/proteolytic and phospholipase activity. On the other hand, lesions induced by *T. nattereri* and *P. fasciatum* venoms are caused independently of the presence of phospholipase proteins. The lack of research aimed at identifying new families of toxins reinforces the importance of this area of study.

**Figure 7.**
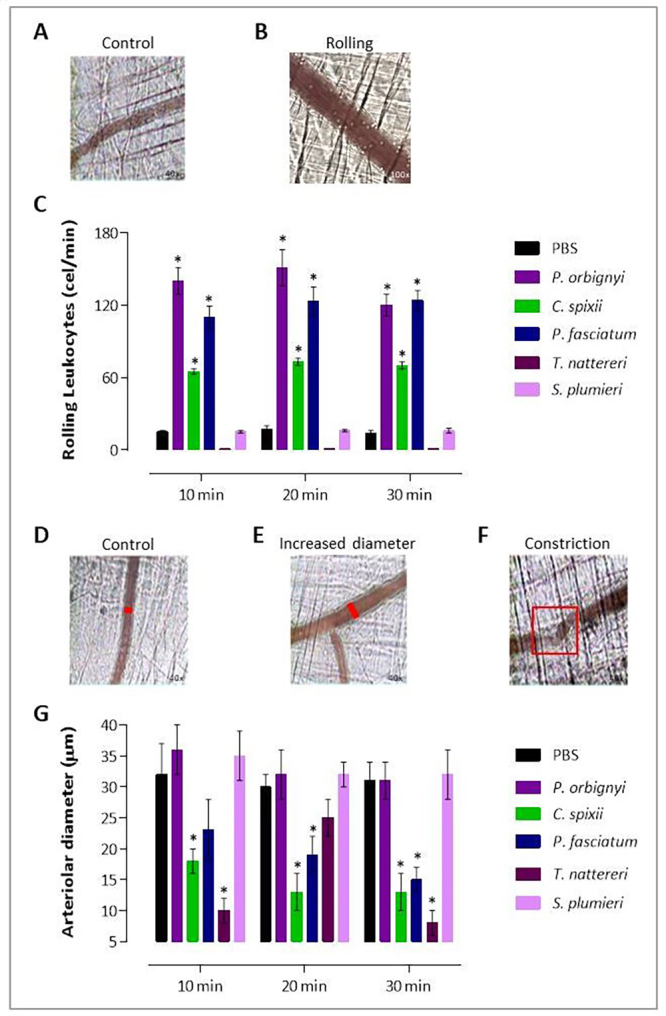
Microcirculatory changes induced by venoms in mice. Cremaster muscle was exposed to a topical application of 20 (L containing 10 (g of each venom in sterile PBS for evaluation of rolling leukocytes and arteriolar diameter. **(A)** Negative-control mice were submitted to topical application of sterile PBS. **(B, C)** Rolling leukocytes (velocity of 5 (m/s) were counted at 10, 20 and 30 min in post-capillary venules of venom injected mice. **(D-G)** The diameters of the arterioles compared with negative control resulted in outcomes like increased or constricted arterioles. Bars represent the mean of 5/group ± SEM. * *p* < 0.05 in relation to negative-control group.

Intravital imaging approaches in semitransparent tissue structures such as cremaster muscle examined microvascular damage at very high magnification characterized by arteriolar vasoconstriction/dilatation and hyper-contraction of muscle fibers and neutrophil dynamics in post-capillary venules [58, 59]. Thus, we used this technique to reveal the initial steps of the leukocyte recruitment cascade, such as leukocyte-vessel wall interactions translated into rolling leukocytes (Figure 7 A and Figure 7 B ). Arteriole diameters such as standard caliber (Figure 7 D), increased (Figure 7 E ), and constriction (Figure 7 F ) were determined. We observed that *P. orbignyi* venom provoked the highest intensity of rolling leukocytes, with 140 ± 25 at 10 min, 151 ± 28 at 20 min, with a decrease to 120 ± 22 in 30 min compared to negative control with 15 ± 1 at 10 min, 17 ± 1 at 20 min, and 14 ± 1 at 30 min (p < 0.05). *P. fasciatum* venom also induced high and increasing levels of rolling leukocytes (110 ± 22, 123 ± 25, and 124 ± 21), followed by *C. spixii* venom that induced the lowest level of rolling leukocytes (65 ± 4, 73 ± 8, and 70 ± 5). Interestingly, the venoms of *S. plumieri* and *T. nattereri* did not mobilize leukocytes in the vessels of mice compared to the negative control (Figure 7 C ).

Small resistance arteries and arterioles constrict to maintain homeostasis in the cardiovascular system by controlling pressure and blood flow. However, with the persistence of vasoconstriction, ischemic hypoxia due to perfusion deficiency occurs [60]. Mechanical stresses sensed by various receptors on vascular smooth muscle cells (VSMCs) induce signals that lead to vasoconstriction [61, 62].

We showed that *C. spixii* venom induced a persistent arteriole contraction (44%, 59%, and 58%) compared to the control group. The *T. nattereri* venom also induced a transient decrease in arteriole diameter by 69% at 10 min and 74% at 30 min. The venom of *P. fasciatum* induced, from just 20 min, a decrease in the diameter of arterioles (41% at 20 min and 52% at 30 min). Interestingly, the venoms of *P. orbignyi* and *S. plumieri* did not induce arteriolar constriction (Figure 7 G ).

**Figure 8.**
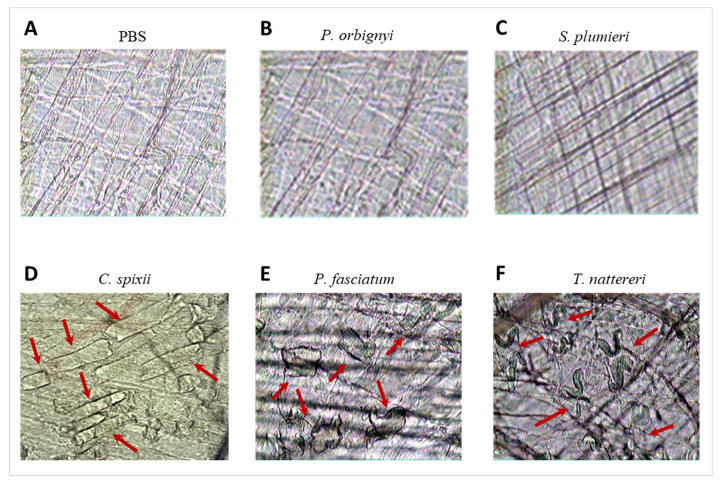
Venoms induced myofibrillar hyper-contraction seen through representative photomicrographs of muscles of mice. Cremaster muscle of mice was exposed to a topical application of 20 (L containing 10 (g of each venom in sterile PBS. **(A)** Negative-control mice were submitted to topical application of sterile PBS. **(B, C)** Absence of changes was observed after topical application of PBS in negative control mice or after *Potamotrygon orbignyi* or *Scorpaena plumieri* venoms. **(D-F)** Hyper-contraction indicated by red arrows was observed in myofibrils after 10 min of application of *Cathorops spixii*, *Pseudoplatystoma fasciatum*, and *Thalassophryne nattereri* venoms.

Ischemia induces oxidative stress and prompts the accumulation of intracellular sodium, hydrogen, and calcium ions, reaching tissue acidosis. The consequences are myofibrillar hyper-contractility, adenosine triphosphate depletion, and ultrastructural injury to mitochondria [63]. Finally, herein when the muscle fibers were analyzed, we observed that the venoms of *P. orbignyi* (Figure 8 B ) and *S. plumieri* (Figure 8 C ) were not able to induce alterations compared to negative control mice, while the venoms of *C. spixii* (Figure 8 D ), *P. fasciatum* (Figure 8 E) and mainly of *T. nattereri* (Figure 8 F ) directly affected the integrity of skeletal muscle plasma membrane inducing intense myofibrillar hyper-contraction compared with each other and with the normal muscles of the negative control group (Figure 8 A ). 

**Figure 9.**
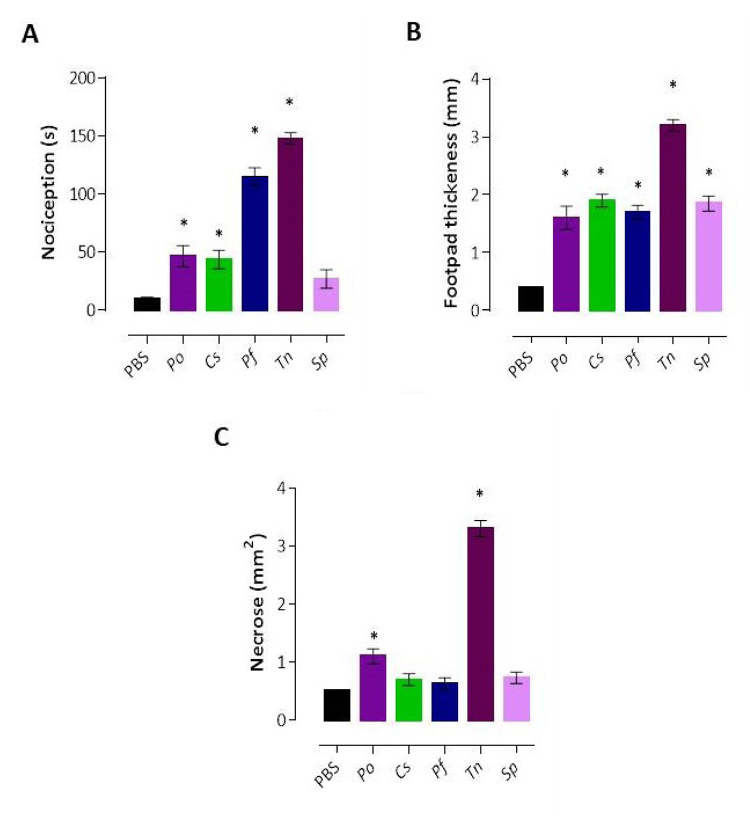
Comparison of the main local toxic effects induced by fish venoms in mice. **(A)** Nociception activity was induced in mice by an intraperitoneal injection of venom, administered at a dose of 30 (g in 30 (L of PBS, into the right hind paw. Each mouse was kept for 30 min under observation of the time spent, in seconds, licking or biting the injected paw. **(B)** Edema was quantified by measuring the thickness of injected paws with a pachymeter after 2 h of venom injection. The results were presented by the difference between experimental and control footpad thickness. **(C)** For necrosis evaluation, 50 (g of venom in 50 µL of PBS was intradermal injected into the shaved backs of mice. After 72 h, they were killed and skin removed for necrotic area measurement. For all evaluations, mice injected only with sterile PBS were considered a negative control group. Bars represent the mean of 5/group ± SEM. *p < 0.05 compared to the control group. (Po) *Potamotrygon orbignyi*, (Cs) *Cathorops spixii*, (Pf) *Pseudoplatystoma fasciatum*, (Tn) *Thalassophryne nattereri*, (Sp) *Scorpaena plumieri*.

Symptoms of fish envenomation resulting from damage to microcirculation and muscle fibers include swelling, pain, and difficult-to-heal lesions. Pain may be due to several factors, including the complex interaction between mediators produced by inflammatory cells and sensitization of peripheral nociceptors by toxins [64, 65, 66]. First, we compared the pain induced by the venoms when applied at the same dose of 30 µg in Swiss mice monitored for 30 min. Figure 9 A shows that the *T. nattereri* and *P. fasciatum* venoms induced the highest nociceptive response (147.7 ± 5 and 114.7 ± 8, respectively, compared to 10 ± 1 of the negative control (p < 0.05). *P. orbignyi* and *C. spixii* venoms induced similar levels of nociception (46.4 ± 9 and 43.5 ± 8, respectively) that were three times lower than *T. nattereri* venom and 2.5 times lower than *P. fasciatum* venom. On the other hand, *S. plumieri* venom did not induce the nociceptive response at this dose.

Fluid accumulation leading to tissue edema, as it occurs in fish envenomation, is associated with the extravasation of plasma proteins through interendothelial gaps [67]. When we compared edematogenic response using each of the venoms at 30 µg (Figure 9 B ), the venoms of *P. orbignyi*, *C. spixii*, *P. fasciatum*, and *S. plumieri* were able to induce similar edematogenic responses with moderate level (1.6 ± 0.2, 1.9 ± 0.11, 1.7 ± 0.12, and 1.85 ± 0.13) compared to negative control (0.4 ± 0.01; p < 0.05) in contrast to the venom of *T. nattereri* which induced the highest edematogenic response (3.2 ± 0.1).

Muscle necrosis is a probable clinical complication of fish envenoming, which in critical cases can lead to functional or physical long-term dysfunctions. The comparison of the ability of venoms to induce necrosis was evaluated in mice injected with venoms at 50 µg (Figure 9 C). *T. nattereri* venom showed the highest necrotic activity (3.3 ± 0.14) compared with each other and with negative control (with 0.5 ± 0.01, p < 0.05). Although the venom of *P. orbignyi* presented this capacity, the necrosis was three times smaller than that of toadfish venom, with an area of 1.1 ± 0.13. However, the venoms of both catfish and scorpionfish could not induce necrosis.

We demonstrated that the venom of *C. spixii*, as well as the venoms of *P. orbignyi*, *T. nattereri*, and *P. fasciatum* present potent toxicity, causing severe local response in contrast to the venom of *S. plumieri* which, in addition to inducing only a moderate level of edema, it is unable to cause nociception or necrosis, corroborating data that show its ability to cause systemic manifestations such as acute cardio-respiratory syndrome preferentially [13].

The action of fish toxins, including proteinases, causing membrane rupture can lead to the release of damage-associated molecular patterns (DAMPs). These DAMPs will act on multiple receptors and signaling pathways, generating a hyperinflammatory and hypercoagulable condition. As a result, platelets, endothelial, and innate cells will be activated, and oxidative-inflammatory and thrombotic events will be triggered [68].

The natterin family of proteins activates sentinel cells, epithelial and endothelial cells to promote the release of the alarmin IL-33 [69], and catfish venom induces mast cell-dependent lipidic mediators’ production [14, 15]. In response to this initial inflammatory stimulus, a new wave of inflammatory innate cytokines (TNF-(, IL-6, and IL-1() is produced, as well as the activation of MMP-2 and MMP-9 activity that affects post-capillary venules, creating an adhesive surface to attract leukocytes such as neutrophils within the affected tissue, exacerbating tissue damage. Stingray envenomation provoked by toxins produced by glands dispersed along the stingers, which are covered by mucus, is characterized by an acute inflammatory response independent of mast cells but dependent on the IL-33 and ST2 signaling axis [28].

Therefore, the effects of *C. spixii*, *P. fasciatum*, and *T. nattereri* venoms inducing structural microvascular alterations may predict the development of target organ lesions and complications such as total vascular events, including cardiovascular events [70]. Pore-forming toxins such as natterin, in addition to acting on microvascular hemodynamics, are also capable of altering mitochondrial oxidative metabolism [71], described as one of the critical initial causes of ischemic necrosis [72, 73] and activate the inflammasome complex, releasing mediators that drive cellular senescence in the lesion [43].

## Conclusion

Improving health outcomes and safety for artisanal fishers in coastal regions of Brazil is a challenge that still requires collaborative efforts from public health authorities, researchers, stakeholders, and the community. By integrating these measures, we can mitigate the impact of venomous fish encounters and ensure better health outcomes for vulnerable populations.

The survey data gathered here highlight the need for a more standardized approach to the management of venomous fish encounters in Brazil, suggesting that the current lack of a unified protocol is a gap in the literature and existing medical practices. Furthermore, underreporting and the lack of comprehensive data on venomous fish encounters in these regions highlight the need for improved reporting systems and data collection methods to accurately assess the scope and severity of the problem.

New strategies are needed to manage fish envenomation, including expanding data on the pathophysiology of lesions caused by different species in Brazil, identifying typical fish toxins responsible for toxic effects, applying experimental models to verify the therapeutic effect of different classes of drugs, creating a database on accidents in Brazil and the symptoms presented by patients, promoting follow-up studies and evaluation of patients to obtain a better stratification of systemic risks and, possibly, the availability of specific therapeutic measures, such as polyspecific antivenom therapy.

Here, we provide the first comparative portrait of the toxic activities of Brazilian fish venoms. Our results show a hierarchy in the venom’s ability to induce local toxic effects in mice, probably related to the diversity of toxic compounds with specific toxins of single species.

The diversity of toxins in fish venoms underlies the different lesion and symptom profiles. The identification of genes and toxins represents an important goal of current studies of venomous fish, and this requires the integration of several methods. Multiomics integration may become a tool to build a comprehensive causal relationship between toxin signatures and the phenotypic manifestations of envenomation caused by fish venoms. Furthermore, the identification of new families of toxins in these venoms will provide insights into mechanisms of action and therapeutic targets.

In conclusion, specifically for antivenom therapy, knowledge of the hierarchy of toxic activities of the most clinically relevant fish venoms may provide support for the best antigen formulation for its production.

## Availability of data and materials

 All data generated or analyzed during this study are included in this article. Additional information is available from the corresponding author upon reasonable request.

## Supplementary material

The following online material is available for this article:

Additional file 1. Information about Brazilian venomous fish species. Data on the venom apparatus of each species, popular name, family, order, class, habitat as well as distribution in Brazil and South America.
