# Supplementary material for: A comprehensive overview of fish envenomation and venom toxicity in Brazil
Source: J Venom Anim Toxins Incl Trop Dis. 2025 May 19;31:e20240061. doi: 10.1590/1678-9199-JVATITD-2024-0061 (PMC12088643; doi:10.1590/1678-9199-JVATITD-2024-0061)
Supplement: Additional file 1. [file 1678-9199-jvatitd-31-e20240061-s1.pdf]

J Venom Anim Toxins incl Trop Dis
https://doi.org/10.1590/1678-9199-JVATITD-2024-0061

Supplementary Material to "A comprehensive overview of fish envenomation and venom toxicity in Brazil"

Additional file 1. Information about Brazilian venomous fish species. Data on the venom apparatus of each species, popular name, family, order, class, habitat as well as distribution in Brazil and South America.

Species	*Thalassophryne nattereri*	*Scorpaena plumieri*	*Potamotrygon orbignyi*	*Cathrops spixii*	*Pseudoplatystoma fasciatum*
**Venom apparatus**	Two dorsal and two lateral spines	Thirteen dorsal, three anal and two pelvic short and thick spines	One or two spines bilaterally retro-serrated covered by a tissue layer containing venom glands	Quite rigid bone structure covered by a thin tegument membrane	Quite rigid bone structure wrapped up for a slight tegument membrane
**Popular name**	Toadfish	Scorpionfish	Stingray, ray	Catfish	Catfish
**Family**	Batrachoidae	Scorpaenidae	Potamotrygonidae	Ariidae	Pimelodidae
**Order**	Batrachoidiformes	Scorpaeniformes	Rajiformes	Siluriformes	Siluriformes
**Class**	Osteichthyes	Osteichthyes	Chondrichthyes	Osteichthyes	Osteichthyes
**Habitat**	Near shore areas to estuarine and deep waters	Widespread in tropical and temperate seas	Freshwater with a deep, sandy substrate	Mainly in estuarine and freshwater habitats from Central and South America	Freshwater habitats
**Distribution**	North and Northeast Brazilian coast	Brazilian coast	River systems of South America that drain into the Atlantic Ocean	Southeastern coast of Brazil	South American rivers
